# Management of Cushing’s Disease in Pregnancy: A Case Report

**DOI:** 10.7759/cureus.105582

**Published:** 2026-03-21

**Authors:** Gargi Mukherjee, Shilpa Manmathan, Sunil Zachariah, Jean Arokisamy, Sumit Kar

**Affiliations:** 1 Obstetrics and Gynecology, Surrey and Sussex Healthcare NHS Trust, Redhill, GBR; 2 Diabetes and Endocrinology, Surrey and Sussex Healthcare NHS Trust, Redhill, GBR

**Keywords:** cushing’s disease, gestational diabetes mellitus (gdm), gestational hypertension, high-risk pregnancy, medical disorders in pregnancy, multidisciplinary teams

## Abstract

Cushing’s syndrome (CS) is a rare condition encountered in pregnancy, and Cushing’s disease (pituitary-dependent CS) represents an even smaller subset. The occurrence of pregnancy in women with CS is uncommon because hypercortisolism suppresses the hypothalamic-pituitary-gonadal axis, frequently leading to anovulation and infertility. When conception does occur, Cushing's disease can significantly complicate the course of pregnancy, posing serious risks to both the mother and fetus. Reported maternal complications include hypertension, gestational diabetes mellitus, pre-eclampsia, heart failure, and increased susceptibility to infections, while fetal complications include growth restriction, preterm birth, and intrauterine demise.

We present a case of a 33-year-old woman with a long history of non-specific symptoms, later confirmed to be due to Cushing’s disease secondary to a pituitary microadenoma. Remarkably, she conceived spontaneously before undergoing definitive therapy. Throughout pregnancy, she was managed conservatively under close multidisciplinary supervision, involving endocrinology, obstetrics, and neonatology teams. The optimal management of CS during pregnancy remains controversial, as therapeutic decisions must balance the risks of uncontrolled hypercortisolism against the potential harms of surgical or medical interventions during gestation. In this case, given the stability of maternal and fetal status, a conservative approach was chosen. Despite the challenges posed by physiological changes in pregnancy and the elevated risks associated with CS, the patient’s pregnancy progressed without major complications. She ultimately delivered a healthy term infant via planned elective cesarean section. This case highlights the critical importance of individualized, patient-centered care and reinforces the value of multidisciplinary collaboration in managing such high-risk pregnancies. It also suggests that, in carefully selected patients, conservative management can be a safe and effective strategy, leading to favorable maternal and neonatal outcomes.

## Introduction

Cushing’s syndrome is a rare endocrine disorder caused by chronic exposure to excessive cortisol. It is broadly classified into adrenocorticotropic hormone (ACTH)-dependent and ACTH-independent forms. ACTH-dependent Cushing’s syndrome results from excessive ACTH secretion, stimulating the adrenal glands, most commonly due to a pituitary adenoma (Cushing’s disease), and less frequently from ectopic ACTH production by non-pituitary tumors. In contrast, ACTH-independent Cushing’s syndrome arises from autonomous cortisol secretion by adrenal adenomas, carcinomas, or adrenal hyperplasia. Hypercortisolism suppresses the hypothalamic-pituitary-gonadal axis, leading to anovulation and infertility. Consequently, pregnancy in women with Cushing’s syndrome is rare, with fewer than a few hundred cases reported worldwide, predominantly described in case reports and small case series. Notably, adrenal-origin Cushing’s syndrome appears to be the most common subtype associated with pregnancy, accounting for approximately 60% of reported cases [1].

The impact of Cushing’s syndrome on pregnancy depends on the severity of hypercortisolism. Uncontrolled maternal hypercortisolism is associated with substantial morbidity. Reported maternal complications include gestational hypertension, pre-eclampsia, diabetes mellitus, opportunistic infections, and an increased rate of cesarean delivery [2]. Fetal risks are equally concerning, encompassing miscarriage, intrauterine growth restriction, preterm birth, neonatal infection, hypoglycemia, respiratory distress, and increased perinatal mortality [3]. Both fetal loss and overall morbidity tend to be higher in the presence of active disease [4].

Diagnosis of Cushing’s syndrome during pregnancy is particularly challenging. Many of the physiological changes of normal pregnancy, such as skin pigmentation, central weight gain, and striae, can mimic features of hypercortisolism. Biochemical confirmation is also difficult because pregnancy stimulates the hypothalamic-pituitary-ovarian axis, elevating cortisol levels, which limits the reliability of standard diagnostic tests, including serum cortisol, urinary free cortisol, and dexamethasone suppression testing. Since circadian rhythm is generally preserved, late-night salivary cortisol, using pregnancy-specific cut-off values, is considered the most useful diagnostic tool [4,5].

Management of Cushing’s syndrome in pregnancy remains controversial. Both medical and surgical therapies carry risks to mother and fetus, and treatment must be individualized according to gestational age, disease severity, and available expertise. Surgical intervention is typically preferred in severe cases, with the second trimester regarded as the safest window, although supporting evidence is limited [4]. When surgery is not feasible, medical therapy may be used for disease control, though agents such as cabergoline, ketoconazole, and metyrapone are not routinely approved in pregnancy [4]. However, in cases of mild disease, characterized by modest hypercortisolemia and absence of significant complications, conservative management may be an appropriate option. In such situations, close clinical surveillance is essential, with proactive management of potential complications of hypercortisolism, including optimization of glycemic control, correction of electrolyte imbalance, and careful blood pressure monitoring and treatment [6].

Multidisciplinary management involving endocrinology, obstetrics, anesthesia, and neurosurgery is essential to optimize outcomes in this high-risk setting. Here, we report a rare case of Cushing’s disease due to a pituitary microadenoma, diagnosed prior to conception in a patient who achieved spontaneous pregnancy. This case highlights the diagnostic challenges, therapeutic considerations, and clinical outcomes associated with Cushing’s syndrome in pregnancy, underscoring the importance of coordinated multidisciplinary care and individualized management to optimize both maternal and fetal prognosis.

## Case presentation

A 33-year-old woman was reviewed in the obstetric endocrine clinic in early pregnancy with a long and complex history of Cushing’s disease. Her symptoms began at the age of 18-19 years while she was residing in Mauritius. She reported progressive and severe fatigue, persistent headaches, and rapid weight gain, with an increase in body weight from 50 kg to 120 kg over an eight to nine-month period. She experienced a year of secondary amenorrhea, followed by marked oligomenorrhea, with only two to three menstrual cycles over the subsequent 12 months. During this time, she also developed borderline hypertension and dyslipidemia. In addition, she described extensive violaceous striae, the prominence of which fluctuated over time.

She was initially diagnosed with polycystic ovary syndrome (PCOS), and management focused on lifestyle modification and symptomatic treatment of her blood pressure and lipid abnormalities. Initial biochemical investigations, including thyroid function tests, prolactin, follicle-stimulating hormone (FSH), and luteinizing hormone (LH), were within normal limits. Despite her ongoing symptoms and repeated requests for further evaluation, concerns were initially attributed to PCOS. Owing to the persistence and progression of symptoms, further investigations were eventually undertaken. The results of a battery of tests for Cushing's syndrome (done outside the United Kingdom) are tabulated below (Table 1).

**Table 1:** The battery of tests for Cushing’s syndrome. ACTH: adrenocorticotropic hormone

Tests	Results	Normal range
Low-dose dexamethasone suppression test - cortisol level (nmol/L)	109	<50
ACTH (pg/mL)	50	<46
24 h free cortisol (nmol/24 h)	185	<270

The dexamethasone suppression test demonstrated failure of cortisol suppression following dexamethasone administration. Subsequent contrast-enhanced magnetic resonance imaging (MRI) of the brain identified a 7 mm pituitary microadenoma. This part of her treatment took place outside the United Kingdom, and we were therefore unable to obtain a detailed account of the clinical events, investigation results, or imaging findings. We relied primarily on the history provided by the patient, and only the limited documentation she was able to provide has been included.

Following this diagnosis and series of events, she relocated to the United Kingdom, where she underwent further evaluation and management. On review in the endocrine clinic, her ongoing symptoms included significant weight gain, mild headaches, and oligomenorrhea, with menstrual cycles ranging from 35 to 90 days. Her blood pressure at presentation was within the normal range (120/85 mmHg).

Comprehensive biochemical investigations were undertaken. Baseline assessments included renal function tests, lipid profile, and glycated hemoglobin (HbA1c). Pituitary and reproductive function were evaluated with thyroid function tests, prolactin, follicle-stimulating hormone (FSH), luteinizing hormone (LH), estrogen, testosterone, sex hormone-binding globulin (SHBG), and insulin-like growth factor-1 (IGF-1). Evaluation of adrenal function included repeat dexamethasone suppression testing, 24 h urinary free cortisol, late-night salivary cortisol, and plasma adrenocorticotropic hormone (ACTH) levels. Cross-sectional imaging with abdominal-pelvic ultrasonography and MRI was also performed. Some of the tests were repeated within a few months, and the results are tabulated below (Tables 2, 3).

**Table 2:** Results of investigation for Cushing's disease. ACTH: adrenocorticotropic hormone

Tests	Normal value	April	June
Low-dose dexamethasone suppression test (cortisol at 9 am) (nmol/L)	<50	345	432
24 h urinary cortisol (nmol/24 h)	124	53	147
ACTH (pg/mL)	<50	55	89
Salivary cortisol (at night) (nmol/L)	2.6	-	9.3

**Table 3:** Results of battery of tests with reference range. TSH: thyroid-stimulating hormone; FSH: follicle-stimulating hormone; LH: luteinizing hormone; SHBG: sex hormone-binding globulin; IGF-1: insulin-like growth factor-1

Tests	Result	Normal range
TSH (mIU/mL)	2.5	0.4-4.0
Prolactin (ng/mL)	18.6	4.8-23.3
FSH (mIU/mL)	6.6	3.5-12.5
LH (mIU/mL)	4.2	1.9-12.5
Estrogen (pg/mL)	66	15-350
Testosterone (ng/mL)	33	15-70
SHBG (nmol/L)	24	18-144
IGF-1 (ng/mL)	231	114-492

**Figure 1:**
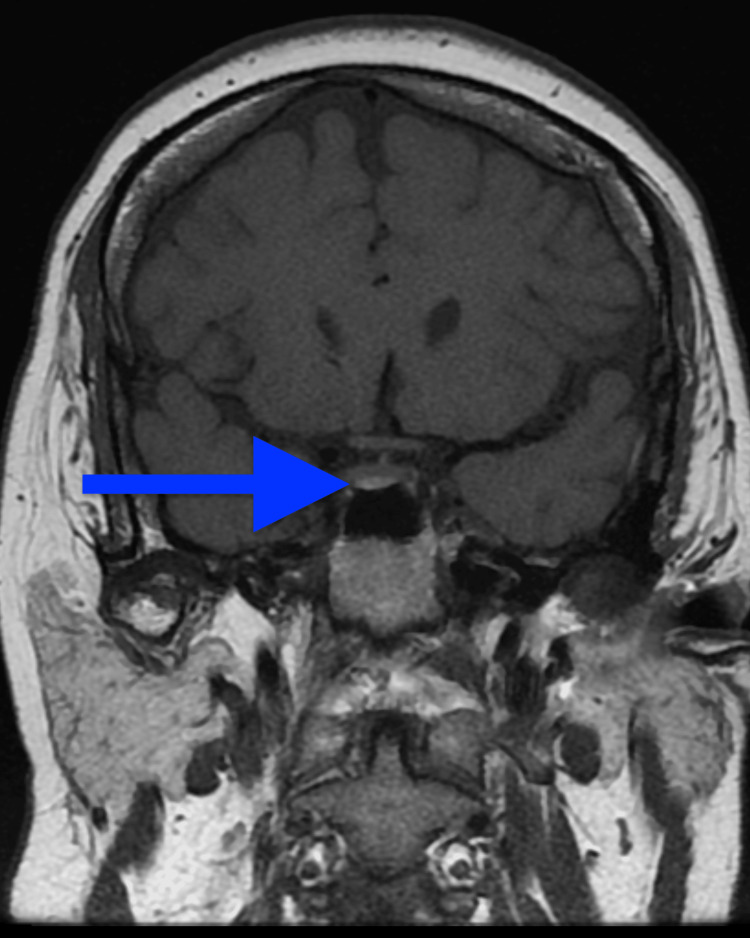
Pre-contrast MRI - sagittal section showing microadenoma (blue arrow).

**Figure 2:**
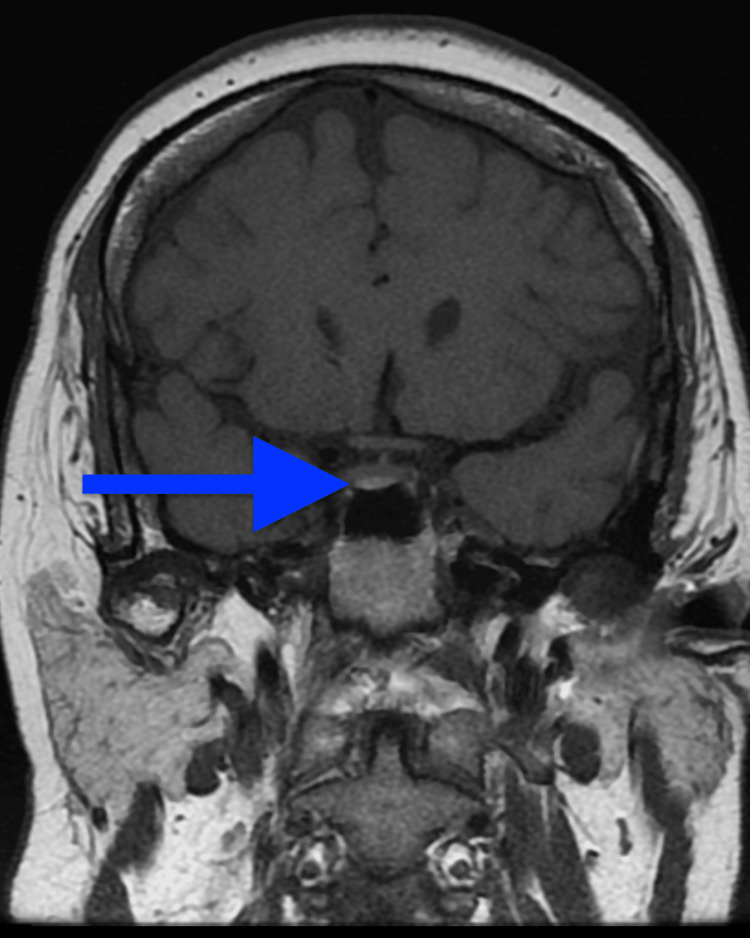
Post-contrast MRI - sagittal section of the pituitary microadenoma (blue arrow).

Her renal function and HbA1c were normal. The two results of failure of the dexamethasone suppression test (raised cortisol), one result of raised 24 h urinary cortisol, and raised salivary cortisol confirmed the diagnosis of Cushing’s syndrome. The imaging showed normal pelvic organs, but the MRI confirmed the presence of a 7 mm right-sided pituitary microadenoma with no involvement of the cavernous sinus or optic chiasm (Figures 1, 2). The imaging confirmed the diagnosis of Cushing's disease. She was referred to neurosurgeons and planned for volume flair/dynamic post-gadolinium (GAD) MRI.

While awaiting further imaging and specialist referral, she conceived spontaneously, and further investigations and definitive management were temporarily deferred. At her antenatal booking appointment at 10 weeks of gestation, her body mass index (BMI) was 50 kg/m². Blood pressure, urinalysis, and routine antenatal blood investigations were within normal limits. Given her background, she was urgently referred to the obstetric endocrine clinic and reviewed at approximately 16 weeks’ gestation.

At the time of endocrine review, her predominant symptom was fatigue. She denied headaches or galactorrhoea. Since confirmation of pregnancy, she had gained approximately 3 kg in weight. There were no overt features of Cushingoid facies. She reported extensive striae, which had been purplish in color in early pregnancy but had gradually faded; on examination, the striae were no longer violaceous. Her skin otherwise appeared healthy, with no evidence of easy bruising. Mild proximal myopathy was noted on clinical examination.

At the time of review, her blood pressure was elevated at 150/90 mmHg, although urinalysis remained normal. She was commenced on labetalol 200 mg twice daily, low-dose aspirin 150 mg daily, and prophylactic low-molecular-weight heparin (LMWH). Blood tests demonstrated mild iron-deficiency anemia, with a hemoglobin level of 95 g/L and ferritin of 15 µg/L; iron supplementation was initiated. An oral glucose tolerance test (OGTT) performed at 16 weeks’ gestation was normal.

Given the stability of her symptoms, a conservative management approach was adopted with close surveillance in a joint obstetric-endocrine clinic. The obstetric management plan included regular blood pressure monitoring, screening for pre-eclampsia, and serial fetal growth assessments from 28 weeks’ gestation onwards. A repeat OGTT was scheduled for 24-26 weeks’ gestation. The endocrine plan involved repeat pituitary MRI and clinical surveillance for progression of Cushing’s disease.

Her blood pressure remained controlled on labetalol 200 mg twice daily initially, but increased during the third trimester, necessitating escalation to 200 mg three times daily at 34 weeks’ gestation. Urinary protein remained negative throughout pregnancy. The repeat OGTT at 24 weeks confirmed gestational diabetes mellitus, which was managed with dietary modification and metformin.

Serial fetal growth scans from 28 weeks demonstrated appropriate growth with normal Doppler studies. A 36-week scan confirmed a breech presentation. She was reviewed in the obstetric anesthetic clinic because of her high BMI and complex medical history and was deemed suitable for all forms of anesthesia, although general anesthesia was advised to be avoided if possible.

She remained clinically stable throughout pregnancy. Repeat pituitary MRI performed during the second and third trimesters demonstrated a stable microadenoma with no radiological evidence of progression. Although fatigue persisted, there were no additional clinical features suggestive of worsening Cushing’s disease.

Due to the presence of persistent breech presentation, she opted for an elective cesarean section. The surgery was carried out at 37+5 weeks and was uneventful. The male infant weighed 2.7 kg and cried immediately after birth. The post-operative recovery was also normal.

She was reviewed in the endocrine clinic at 12 weeks postpartum. Her post-operative recovery was uneventful, with no new signs or symptoms. Blood pressure had normalized, allowing for discontinuation of labetalol at four weeks postpartum. Other blood tests, including full blood count (FBC) and HbA1c, were performed at six weeks postpartum and returned within normal limits. However, repeat investigations confirmed elevated 24-h urinary cortisol and ACTH levels. She was subsequently referred back to the neuroendocrine clinic for definitive management.

## Discussion

Cushing’s disease (CD) during pregnancy is a rare clinical entity, with approximately 250 cases documented in the literature [1]. Hypercortisolism is known to disrupt the hypothalamic-pituitary-gonadal axis, leading to ovulatory dysfunction and resulting in reduced fertility. Therefore, conception in the setting of active CD is uncommon. This case contributes to the limited body of evidence on pituitary-dependent Cushing’s syndrome in pregnancy, which was managed conservatively by the multidisciplinary team and had a successful outcome.

When pregnancy occurs in women with Cushing’s syndrome, whether of pituitary or adrenal origin, it is associated with a significantly increased risk of maternal and fetal complications [2,7]. Maternal complications may include gestational diabetes, gestational hypertension, pre-eclampsia, and a higher likelihood of cesarean delivery [1]. Fetal complications include preterm birth, neonatal infections, hypoglycemia, respiratory distress syndrome, and stillbirth. The risk of fetal loss is notably higher in untreated or poorly controlled cases of Cushing’s syndrome. Given these risks, pregnancy is generally discouraged in women with active Cushing’s disease, and conception should ideally be deferred until biochemical remission is achieved.

Routine diagnostic tests for Cushing’s syndrome, such as the dexamethasone suppression test, are generally avoided during pregnancy due to a high false-positive rate. The hypothalamic-pituitary-adrenal (HPA) axis is activated during normal pregnancy, producing increased levels of corticotropin-releasing hormone (CRH) (much of it originating from the placenta), ACTH, and serum total and free cortisol. There is also increased hepatic production of cortisol-binding globulin related to high levels of estrogen, which further increases measured serum cortisol. The levels continue to increase as the pregnancy progresses. Thus, there is a high possibility of false positives in the routine tests used for the diagnosis of Cushing's disease. Twenty-four-hour urinary cortisol also remains high in pregnancy; thus, any value three times higher than normal would indicate Cushing's syndrome. However, late-night salivary cortisol remains a useful screening tool, as diurnal variation in cortisol secretion is relatively preserved in pregnancy. The following cut-off values are used in pregnancy: 0.255 µg/dL (7.0 nmol/L) for the first trimester, 0.260 µg/dL (7.2 nmol/L) for the second trimester, and 0.285 µg/dL (7.9 nmol/L) for the third trimester [4].

In the present case, the patient had a pre-established diagnosis of Cushing’s syndrome secondary to a pituitary microadenoma, and was awaiting gadolinium-enhanced MRI for further evaluation [8]. However, confirmation of pregnancy necessitated postponement of this imaging, as the safety of gadolinium contrast during pregnancy remains uncertain and it is not routinely recommended. Other invasive localization procedures, such as inferior petrosal sinus sampling (IPSS), are avoided due to radiation exposure, procedural complexity, and an increased risk of venous thrombosis during pregnancy [9].

The management of Cushing’s disease during pregnancy is complex and requires a multidisciplinary approach, involving obstetricians, endocrinologists, neonatologists, and, in selected cases, endocrine surgeons. Management decisions are individualized based on gestational age, disease severity, and the underlying source of cortisol excess.

In pregnant women with severe Cushing’s disease, surgical intervention - either transsphenoidal pituitary surgery or laparoscopic adrenalectomy - is considered the first-line treatment, particularly during the second trimester [1,10]. Studies have shown that surgical management can significantly reduce fetal complications, such as miscarriage, preterm delivery, and low birth weight [1].

Medical therapy is reserved for patients who are not surgical candidates (mild to moderate disease, advanced gestation, patient refusal) or as initial therapy for symptom control. The commonly used medications include metyrapone, ketoconazole, and cabergoline. Among these, metyrapone is the most frequently used during pregnancy; however, it is associated with side effects such as worsening hypertension and hypokalemia. The safety data on metyrapone, ketoconazole, and cabergoline in pregnancy are limited, and their use is typically guided by clinical judgment and disease severity.

Patients with Cushing’s disease are at high risk of thromboembolic events during pregnancy and the postpartum period. Therefore, low-molecular-weight heparin (LMWH) prophylaxis is recommended. In the present case, the patient had additional risk factors for thrombosis (raised BMI) and was accordingly managed with LMWH during both antenatal and postnatal periods (up to six weeks postpartum) [4].

An essential component of this patient’s management was close clinical surveillance. Throughout the pregnancy, she remained clinically stable, with no evidence of progression of Cushing’s disease. She did not develop any new classical features of hypercortisolism, such as violaceous striae or a buffalo hump. In addition, there were no symptoms suggestive of pituitary tumor enlargement, including headache or visual disturbances. Serial MRI imaging demonstrated no increase in the size of the pituitary microadenoma. We did not monitor the blood test results since the results were inconsistent (due to associated biochemical changes in pregnancy). The blood pressure and glycemic control were well controlled, and there were no signs of adrenal or cortisol crisis, enabling a conservative, non-surgical approach. Due to associated risks of perinatal morbidity and mortality, increased fetal surveillance is recommended - the patient’s serial growth scans showed normal growth, and the pregnancy progressed to term. Delivery was achieved via elective cesarean section, indicated by breech presentation.

There is currently no clear consensus regarding the optimal timing or mode of delivery, nor the precise management during labor. Vaginal delivery is not contraindicated in itself; however, the mode and timing of birth should be guided by the severity of the disease and the presence of any associated obstetric complications. Intrapartum management should be individualized and carefully planned by a multidisciplinary team to optimize both maternal and fetal outcomes. Since postnatal reassessment confirmed persistent hypercortisolism, the patient was referred for definitive neurosurgical intervention.

This case highlights the critical importance of multidisciplinary collaboration, individualized decision-making, and tailored antenatal surveillance in the management of Cushing’s disease during pregnancy. It further demonstrates that conservative management may be a safe and effective option in select cases with mild disease activity, provided that there is rigorous monitoring and coordinated care.

## Conclusions

This case underscores the significant management challenges posed by Cushing’s disease in pregnancy, a rare condition associated with considerable maternal and fetal morbidity. Achieving favorable outcomes requires an individualized approach, with careful monitoring to assess disease progression and a thoughtful balance between the risks of intervention and the potential consequences of untreated hypercortisolism.

Management options include conservative, medical, and surgical approaches. Patients with mild hypercortisolism like this can be managed conservatively with close clinical and radiological surveillance. Our report highlights the critical importance of a coordinated multidisciplinary approach to optimize both maternal and neonatal outcomes in this complex clinical scenario.
